# The Role of Extracellular Vesicles in Modulating the Host Immune Response during Parasitic Infections

**DOI:** 10.3389/fimmu.2014.00433

**Published:** 2014-09-08

**Authors:** Sergio Montaner, Alicia Galiano, María Trelis, Lorena Martin-Jaular, Hernando A. del Portillo, Dolores Bernal, Antonio Marcilla

**Affiliations:** ^1^Àrea de Parasitologia, Departament de Biologia Cel.lular i Parasitologia, Universitat de València, Burjassot, Spain; ^2^Barcelona Centre for International Health Research (CRESIB, Hospital Clínic-Universitat de Barcelona), Barcelona, Spain; ^3^Institució Catalana de Recerca i Estudis Avançats, Barcelona, Spain; ^4^Departament de Bioquímica i Biologia Molecular, Universitat de València, Burjassot, Spain

**Keywords:** extracellular vesicles, parasite, protozoa, helminth, immunomodulation

## Abstract

Parasites are the cause of major diseases affecting billions of people. As the inflictions caused by these parasites affect mainly developing countries, they are considered as neglected diseases. These parasitic infections are often chronic and lead to significant immunomodulation of the host immune response by the parasite, which could benefit both the parasite and the host and are the result of millions of years of co-evolution. The description of parasite extracellular vesicles (EVs) in protozoa and helminths suggests that they may play an important role in host–parasite communication. In this review, recent studies on parasitic (protozoa and helminths) EVs are presented and their potential use as novel therapeutical approaches is discussed.

## Parasitic Diseases and Extracellular Vesicles

Evidence of parasite infections has been found very early in human evolution. In fact, some parasites were inherited from our primate ancestors in Africa, and some others were acquired from animals during our evolution, migrations, and agricultural practices (1). It is estimated that about 300 species of helminths and over 70 species of protozoa affect humans (1). A relatively small proportion of these parasites cause some of the most important diseases in the world, such as malaria, Chagas’ disease, sleeping sickness, schistosomiasis, filariasis, and soil-transmitted helminthiasis among others. Despite their elevated global prevalence, they are considered as neglected tropical diseases^1^. In endemic areas, epidemiological studies of some immunological disorders (i.e., atopy) suggest that current parasitic infections have a protective effect (2). In contrast, in developed countries, where improved living conditions and vaccination are common, the lack of exposure of our immune system to infections of historical importance, could lead to an increase in hypersensibility and autoimmune diseases.

In the last decades, extracellular vesicles (EVs) have been well recognized as mediators of intercellular communications in prokaryotes and eukaryotes. They are able to carry proteins, lipids, and nucleic acids, which are incorporated by recipient cells, where in turn they have different effects. EVs carry a common group of proteins and also specific proteins that reflect the particular role and/or composition of their cell of origin. EVs include usually apoptotic bodies, microparticles/microvesicles (originated by plasma membrane budding), and exosomes [released from multivesicular bodies (MVBs)] (3, 4). Different EVs have been described in most groups of parasitic protozoa, including flagellates, sporozoa, and microsporidians, and they have been detected in extracellular and intracellular stages. In addition to protozoa, parasitic helminths have been recently shown to release EVs (Table 1).

**Table 1:** **Parasites and EVs**.

		Reference
**PROTOZOANS**		
**Trypanosomatids**
*Leishmania* spp.	Exosomes and EVs from infected macrophages	(5–8)
*Trypanosoma brucei*	Exosomes	(9)
*Trypanosoma cruzi*	Outer membrane-derived vesicles, exosomes	(9–15)
**Apicomplexa (sporozoa)**
*Plasmodium vivax*	Plasma-derived MPs	(16, 17)
*Plasmodium berghei*	Plasma-derived MPs (from infected mice)	(18, 19)
*Plasmodium yoelii*	Plasma-derived exosomes	(20)
*Plasmodium falciparum*	Plasma-derived exosomes and vesicles (60–100 nm) and microvesicles (100–1000 nm)	(17, 21–23)
*Plasmodium malariae*	Plasma-derived exosomes	(17)
*Toxoplasma gondii*	Exosomes from infected cells; exosomes	(24–26)
*Cryptosporidium parvum*	Exosomes from infected cells	(27)
*Eimeria* spp.	Dendritic cells derived exosomes (from infected chickens)	(28, 29)
**Flagellates**
*Trichomonas vaginalis*	Exosomes	(30)
*Giardia duodenalis*	Secretory vesicles, exosomes	(31, 32)
**HELMINTHS**		
**Cestodes**
*Echinococcus multilocularis*	Vesicles derived from metacestodes	(33–35)
**Trematodes**
*Schistosoma* spp.	Shedding vesicles	(36)
*Echinostoma caproni*	EVs; exosomes	(37, 38)
*Fasciola hepatica*	EVs; exosomes	(37, 39)
*Dicrocoelium dendriticum*	Exosomes	(40)
**Nematodes**
*Heligmosomoides polygyrus*	EVs	(41)

*Data were obtained also from Ref. not cited in the text (8, 9, 14, 15, 19, 22, 23, 33–36, 38, 39)*.

With respect to EVs composition, studies in *Leishmania* spp. parasites, the protozoan causing different forms of leishmaniases^2^, have shown the presence of protein homologs to known proteins that regulate exosome biogenesis and release in mammalian cells (42, 43). Recently, Silverman and Reiner have proposed that *Leishmania* are capable of secreting both exosomes and plasma membrane blebs, as mammalian cells do, suggesting that both types could play a role in pathogenesis (5). Most of the studies on EVs composition have been focused on intracellular stages of *Leishmania* spp., and it has been shown that changes in the environment seem to affect vesicle release and cargo (6, 42). In fact, proteomic analysis has revealed that the protein cargo of *Leishmania* exosomes is quantitative different in response to changes in temperature and pH (42). In this context, exosomes obtained at neutral pH were enriched in kinase activity, meanwhile in acidic pH they were enriched in phosphatase activity (42). Similar results were obtained after treating *in vitro* extracellular stages of *Leishmania* with a short heat-shock treatment (6).

Among the proteins identified in *Leishmania* EVs, there are virulence factors like GP63/leishmanolysin, membrane proteins, and redox enzymes like tryparedoxin peroxidase and heat-shock proteins (Hsp) (5). As suggested by Silverman and Reiner, this specific packaging of individual proteins and functional groups may likely reflect a sophisticated packaging of virulence factors by *Leishmania* in response to specific environments (5).

Other intracellular organisms like apicomplexans *Plasmodium* and *Toxoplasma* species have been described to produce EVs.

*Plasmodium* species are the causative agents of malaria, a disease affecting an estimated 207 million individuals^3^. Although previous studies had detected EVs in peripheral blood of *Plasmodium falciparum* as well as *Plasmodium vivax* patients (16–18), the first description of *Plasmodium* spp. exosomes was reported in 2011 by Del Portillo and co-workers (20), who revealed the presence of parasite proteins in reticulocyte-derived exosomes (rex) from experimental infections. These parasite antigens included serine-repeat antigens, merozoite surface proteins 1 and 9, metabolic enzymes like lactate dehydrogenase, GAPDH, enolase and aldolase, cysteine proteases, and Hsp among others (20).

*Toxoplasma gondii* is responsible for toxoplasmosis, an important public health problem infecting about 30% of the world’s population, including immunocompromised individuals (44). *Toxoplasma* is promiscuous and can infect virtually any nucleated host cell (45). The existence of EVs (65 nm) in MVBs, has been detected in *Toxoplasma* secretory organelles (24). Lately, the presence of miRNA in *T. gondii* exosomes has been reported (25).

The kinetoplastida *Trypanosoma cruzi* and *Trypanosoma brucei* are the causal agents of the Chagas’ disease and sleeping sickness, respectively. Chagas’ disease affects 7–8 million people mostly in Latin America^4^, and sleeping sickness threats millions of people in 36 countries in sub-Saharan Africa^5^.

The extracellular phase of *T. cruzi* (trypomastigota) produces EVs that contain surface components like glycoproteins gp85/transialidases, alphaGal-containing molecules, proteases (i.e., cruzipain), cytoskeleton proteins, mucins, and associated to GPI (glycosylphospatidylinositol)-anchored molecules. All these molecules are engulfed by host cells in the absence of parasitic cells, and are accumulated in phagocytic/endocytic compartments (10).

In addition to proteins, the presence of small RNA in EVs from *T. cruzi* has been reported, including tRNA, which were actively secreted to the extracellular medium and acted as vehicle for the transfer of these molecules to other parasites and to mammalian cells (11). Furthermore, EVs secreted by *T. cruzi* epimastigotes are able to induce epigenetic changes in host cells (12).

The extracellular flagellate *Trichomonas vaginalis* is the causal agent of trichomoniases, the most prevalent curable sexually transmitted infection globally (46). This parasite produces EVs to allow its attachment to the host mucosa (30). The proteomic analyses of these EVs revealed that 75% of the identified proteins corresponded to orthologs of mammalian exosome proteomes (exocarta). Common proteins represent core conserved exosomes protein families such as tetraspanins, Alix, Rabs, Hsp70, subunits of heterotrimeric G proteins, and TcTP (30). The identified proteins were sorted into functional groups, and the more abundant corresponded to signaling proteins (14%), metabolic enzymes (14%), cytoskeletal proteins, and proteins involved in transport and vacuolar proteins (30).

When comparing the proteomics profiles of intracellular and extracellular protozoa EVs, it seems that a common pattern of proteins is present in both, which include metabolic enzymes and Hsp. An enrichment in proteins involved in transport and vacuolar proteins is observed in EVs from extracellular protozoa (i.e., tetraspanins).

The diseases caused by helminths are considered the most neglected ones, with a third of the human population affected at least by one species (47). EVs from the trematode species *Echinostoma caproni*, *Dicrocoelium dendriticum*, and *Fasciola hepatica* have been isolated. The analysis of the composition of these vesicles has identified proteins previously described in the excretory/secretory products (ESP) (about 50% of proteins corresponded to the proteins identified in the secretome) (37, 40), which may explain the atypical protein secretion (lacking typical secretion signals) in flukes. These proteins include metabolic enzymes like enolase, GAPDH, aldolase, and well-known exosome components like Hsp70 and annexins (37, 40). Differences in EVs composition were observed among the three species, correlated with their respective ESP. Meanwhile, no proteases were present in *E. caproni* EVs, *D. dendriticum*, and *F. hepatica* EVs contained leucine aminopeptidase (LAP), and *F. hepatica* EVs contained a large number of proteases (i.e., cathepsins), probably related to its migration along tissues (37, 40), as well as chaperons, fatty-acid binding proteins, and detoxifying enzymes (37). In addition to the presence of proteins in helminth EVs, the presence of miRNA in *D. dendriticum* EVs has been described (40).

## Parasitic EVs in Cell–Cell Communication

Parasite EVs participate in parasite–parasite and host–parasite communication processes.

Very little information is available about the role of EVs in intraspecific communications. A recent study has demonstrated that *P. falciparum* infecting red blood cells directly communicates with other parasites using EVs that are capable of delivering genes. Importantly, communication via EVs also promotes differentiation to sexual forms and survival of parasites, providing a mechanism for increasing parasite persistence in times of stress (48). Furthermore, the *P. falciparum* PfEMP1 trafficking protein (PfPTP2), which plays a key role in the traffic to host cells, has been identified. PfPTP2 functions in the release of EVs into the supernatant, implicating *P. falciparum* molecular machinery in intercellular communications (48).

In contrast, there are many examples of parasite EVs involved in host–parasite communication.

Pioneering studies on EVs in trypanosomes described *T. cruzi* shedding vesicles (20–80 nm) in cultured trypomastigotes (13), and recently, a possible association between intensity of shedding and infectivity of different strains has been proposed (47). These authors suggest that these vesicles could be acting as messengers for invasion, somehow preparing the host cell for the incoming trypanosome, which represents a novel mechanism to explain parasite interaction with the host (10, 49).

Various studies have described the isolation of EVs from different *T. cruzi* stages (10, 11, 26, 49). Importantly, the pre-immunization of mice using trypomastigote vesicles induce severe heart pathology with intense inflammatory reaction and higher number of amastigote nests in cardiac tissue (10), indicating the impact of host–parasite communication. After EVs release, these vesicles form a complex with C3 convertase on the parasite surface, stabilizing the enzyme and inhibiting its activity, protecting parasites from complement lysis and increasing parasite survival (50). Interestingly, these vesicles also carry transforming growth factor β (TGFβ), which could promote parasite invasion in the course of infection *in vivo* and *in vitro* (50).

As pointed out by Deolindo et al. (26), the production of EVs by infective stages of *T. cruzi* confirms their role in parasite survival strategies and in cell–cell communication. Supporting this notion, Garcia-Silva et al. (12) have shown that parasite EVs elicited changes in the host transcriptome upon their incorporation in the cells, which include modification of immune responses pathways (12).

Little is known about EVs in *Giardia duodenalis*, an extracellular parasite of the human intestine that causes diarrheal illness, and with high global prevalence^6^. Some authors reported the presence of secretory vesicles in this parasite associated with encystation processes. The process of release of these vesicles has been suggested to occur after fragmentation of large encystation-specific secretory vesicle in small secretory vesicles, followed by exocytosis, but this was not fully demonstrated (31, 32). Because *Giardia* is one of the earliest branching protists, knowledge of the secretory organelle biogenesis that occurs during its differentiation into cysts offers novel insights into the molecular machinery required for the regulation of the protein transport in higher organisms (31, 32). Recently, it has been reported an increase in *G. duodenalis* EVs formation in response to different conditions (i.e., pH changes, presence of bile, etc.), suggesting that these vesicles could provide a mechanism for parasite adaptation to changing environment encountered in the host during the course of the infection (26).

The finding that helminth EVs are internalized by host cells suggests an important role for these vesicles in host–parasite communications (37). It is possible that helminths could send messengers like mRNA or miRNA into EVs to act on host targets. Supporting this notion, the presence of molecules of miRNA in *D. dendriticum* EVs has been reported (40). Preliminary studies have shown that the nematode *Heligmosomoides polygyrus* produce EVs, which alter inflammatory responses in both cultured cells and in a murine model. These findings would explain how these nematode EVs could mediate cross-phylum communication and may help to suppress the host inflammatory response (51).

### Induction of host EVs secretion

Some descriptions of host–parasite communication through EVs in infected cells have been reported, with examples including mainly apicomplexan like *Toxoplasma* (27), *Plasmodium* (16–18), *Eimeria* (28, 29), and *Cryptosporidium* (52) species.

As mentioned above, *Plasmodium* proteins were detected in rex in experimental infections, which confirm that they are taken up by host cells (20). Studies to determine whether these vesicles are constitutively released, or whether they are released during a particular phase in the parasite cycle, have been reported recently (21). They present evidence that reticulocyte microvesicles (RMVs) release increases steadily during the parasite cycle and peaks late during schizogony or shortly thereafter. This pattern of release coincides with the emergence of a prominent vesicular subpopulation of 150–250 nm in the infected red blood cells (iRBCs) preparation. Altogether, these data demonstrate that the peak release of RMVs from iRBCs occurs shortly before egression (i.e., within the last 6–8 h of the parasite asexual cycle) (21).

Another apicomplexan protozoan are *Eimeria* spp., the etiologic agents of avian coccidiosis, a major parasitic disease of poultry (28). EVs from dendritic cells infected with *Eimeria tenella* parasites were shown to protect animals by (a) increasing body weight gain, (b) decreasing feed conversion ratios, (c) reducing fecal oocyst shedding, (d) decreasing intestinal lesions, and (e) reducing mortality compared with animals given parasite Ag alone (28). Similar results were obtained for other *Eimeria* species, suggesting that this protocol is an efficient way of immunizing against other apicomplexans (29).

*Cryptosporidium* species are another example in which intracellular parasites communicate with their host increasing the production of EVs. Cryptosporidiosis is one of the most frequent causes of diarrhea worldwide, affecting immunocompromised and/or immunocompetent patients (53). The occurrence of large-scale outbreaks of human cryptosporidiosis is often attributed to contaminated drinking water (54).

It has been reported that *Cryptosporidium parvum* infection increases luminal release of EVs from the biliary epithelium, probably through TLR4/IKK2-mediated activation of the multivesicular body exocytic pathway (52). Immunogold staining revealed that these microvesicles were positive for the exosome markers CD63 and ICAM-1. Release of EVs involves activation of TLR4/IKK2 signaling through promoting the SNAP23-associated vesicular exocytic process (52). Furthermore, these authors presented evidence that activation of TLR4 signaling stimulates the biogenesis and luminal release of antimicrobial peptide-shuttling EVs. The anti-*C. parvum* activity of apical EVs released from the epithelium may involve direct binding these vesicles to the *C. parvum* sporozoite surface (52). Confocal analyses showed the fusion of these EVs with *C. parvum* sporozoites causing cargo release within the parasite. These results suggest that all extracellular stages of *C. parvum* (sporozoites, merozoites, and microgametocytes) may be vulnerable to EVs binding/targeting, contributing to gastrointestinal mucosal anti-*C. parvum* defense (52).

Extracellular vesicles derived from macrophages infected with *Leishmania mexicana* display unique protein signatures (composition and abundance of many functional families of proteins, such as plasma membrane-associated proteins, chaperones, and metabolic enzymes) (7). *L. mexicana* surface protease GP63 has been identified in EVs from macrophages exposed to parasite promastigotes (7).

## Parasitic EVs as Immunomodulators

Many of the immunomodulatory proteins lack typical secretion signals for delivery to the extracellular environment, so new secretion routes should be active, involving host–parasite interactions at cellular and subcellular levels, which in turn could be related to immunomodulation processes.

The role of exosomes in modulating the immune response was first described in *Leishmania* spp. in 2010 (42). This study also demonstrated that certain factors associated with infection were able to positively regulate the release of exosomes and modulate their composition (42). A recent study has shown that *Leishmania* parasites mutants lacking the metalloprotease GP63 have a reduced modulatory capacity in relation to wild-type parasites in animal models, and they have also described that exosomes are involved in recruiting neutrophils exhibiting stronger pro-inflammatory properties than the neutrophils recruited by parasites (55).

Trypanosomes also constitute a good example of immunomodulation. Proteins like phosphoglycerate mutase, enolase, pyruvate kinase, and phosphoglycerate kinase, known to be involved in immunosuppressive activity in other organisms, have been also found in the *T. brucei* secretome, suggesting a similar role in infection. *T. brucei* releases a higher amount of proteins in EVs than using a classical secretory pathway, which suggests that *T. brucei* may deliver an avalanche of new epitopes to overwhelm the host immune system, or to communicate between trypanosomes themselves. This is achieved by exchanging receptors in their cytosolic form, which may represent an important survival strategy at the population level (9). Several proteins secreted by *T. brucei* are also detected in *Leishmania* and *T. cruzi* secretomes, suggesting that they are the result of an active and common secretion process (49).

They are interesting immunomodulation studies in malaria. When mice were immunized with *Plasmodium yoelii* purified rex, an increase in the reticulocytemia and in the production of IgG antibodies capable of recognizing *P. yoelii* iRBCs was observed. Remarkably, in combination with the adjuvant CpG oligodeoxynucleotide, rex from an experimental infection with a *P. yoelii* reticulocyte-prone non-lethal strain conferred full and long-lasting protection upon immunization and lethal challenge. Thus, these data show, for the first time, that rex can be explored as a new vaccine against malaria (20).

Furthermore, Mantel et al. have demonstrated that the release of RMVs from iRBCs can activate the pro-inflammatory cytokines interleukin-6 (IL-6), IL-12, and IL-1b, as well as the anti-inflammatory cytokine IL-10, in a dose-dependent manner (21). They also showed that neutrophils pre-incubated with RMVs from uninfected red blood cells (uRBCs) migrated at a slower rate compared these pre-incubated with RMVs from iRBCs or untreated controls. Together, these data demonstrate that RMVs from iRBCs, but not from uRBCs, can strongly stimulate cells of the innate immune system (21).

Extracellular parasites could use exosomes to deliver proteins and/or RNA to manipulate host cell responses, while remaining in the extracellular space, generating important changes in both host immune response and parasite attachment to host cells (30). In this context, *T. vaginalis* may use exosomes to manipulate host defense responses, similarly to the secretion of virulence factors and vesicles by pathogenic bacteria (30). By reducing the IL-8 expression of host ectocervical cells, *T. vaginalis* exosomes may be playing an important role in the establishment of a chronic infection. These EVs may lead to the regulation of IL-6 and IL-8 secretion, preparing and facilitating colonization of the urogenital tract (30).

Helminthiases are parasitic diseases with a high prevalence, which reflects their ability to manipulate the host immune system, preventing parasite expulsion. Helminths are interesting organisms to study immunomodulation, since host immunity has also developed mechanisms to limit their pathology and the ensuing injury, as in some cases, their elimination originates even worse collateral damage.

**Figure 1:**
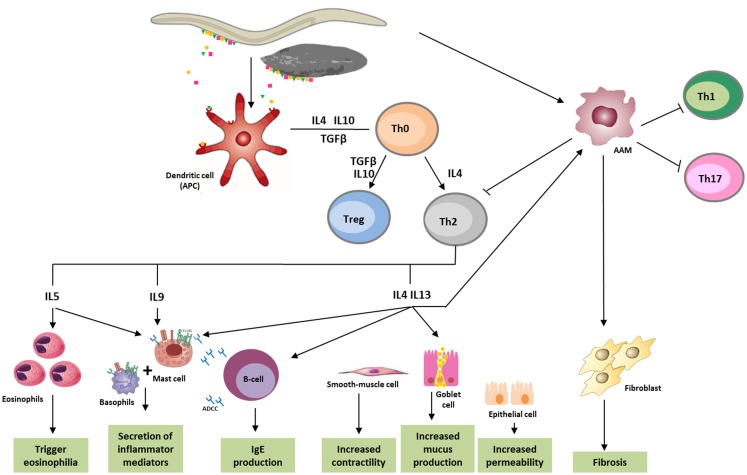
**Immune response against helminths**. Helminth infection mainly leads to Th2 response, involving immune system cells and cytokines. Parasite antigens are recognized by dendritic cells, which in turn act as antigen-presenting cells (APC) for T cells, initiating parasites expulsion. Releasing of cytokines like IL-5, which triggers eosinophilia, and IL-4, IL-9, IL-13, as well as IgE, which bind to the FceRI (high-affinity Fc receptors for IgE), lead to the activation of basophils and mast cells, and cause secretion of inflammatory mediators. IL-4 and IL-13 increase smooth-muscle-cell motility, stimulates intestinal permeability, and elevate mucous secretion by globet-cells. These cytokines also promote the differentiation of alternatively activated macrophages (AAM), which in turn, can inactivate the production of Th1, Th2, or Th17 cells, and in some cases, like in schistosomiasis, induce fibrosis in tissues. ADCC, antibody dependent cellular cytotoxicity; APC, antigen-presenting cells; DC, dendritic cells; AAM, alternatively activated macrophages.

Immune responses to helminths comprise a combination of both innate defense and Th2 response, which disable, degrade, and dislodge the parasites (56). Characteristic features of helminth infection are Th2-dominated immune responses and Th1/Th17 immunity blocking, allowing for the survival of the parasite in a “modified Th2 environment” (57, 58) (Figure 1).

The immune regulation originated by helminths may offer new routes to treat immune dysfunctions like allergy, autoimmunity, and inflammatory bowel diseases (58, 59). Various clinical trials that use helminths to treat autoimmune diseases are underway (60). Furthermore, enhanced allograft tolerance with helminth infection has been reported in various species, suggesting that the infection or defined products from immunomodulatory helminths could be of interest in future transplantation protocols (61). Much research has focused on ESP released by live helminths, which can interfere with every aspect of host immunity. *Schistosoma* species secrete proteins capable of activating the release of IL-4 and activate the degranulation of basophils (human and mice) to promote a Th2 response in the surrounding environment. In addition, *Schistosoma* spp. eggs secrete among others, the protein omega-1, an abundant ribonuclease associated with egg transit through host tissues, and responsible for activating Th2 mechanisms that allow for egg survival and excretion. Furthermore, extensive glycosylation of some *Schistosoma* proteins also trigger Th2 responses *in vivo* through TLR4 ligation (57, 62).

Recent studies have shown that antigens derived from *F. hepatica* tegument inhibit mast cells, which normally play a protective role during microbial infections. This modulating effect is mediated by the induction of suppressors of cytokine signaling (SOCS), which are essential for self-regulatory inflammatory Th1-dependent processes (63).

Interestingly, other helminths produce molecules that have a cytokine-like effect on mammalian cells. Proteins like the macrophage migration inhibitory factor (MIF) are produced by the nematode *Brugia malayi* (57). This parasitic molecule can synergize with IL-4 to induce the development of fully suppressive, alternatively activated macrophages *in vitro*. Thus, in a Th2 environment, parasitic MIF may prevent the classical pro-inflammatory activation of macrophages (57).

As reviewed by Dalton et al. (64), *F. hepatica* ESP includes molecules that drive the immune response toward a favorable, non-protective, Th2-mediated environment. These immunomodulatory molecules include cathepsin L, peroxiredoxins, and helminth defense molecules (i.e., HDM-1/MF6p), which could help treat autoimmune diseases and chronic inflammation in humans and animals (64). Interestingly, two of these proteins (peroxiredoxins and cathepsins) are present in *F. hepatica* EVs (37), and the third type of immunomodulating molecules (HDM-1/MF6p) have been detected in exosomes from the related trematode species *D. dendriticum* (40), and preliminary results suggest their presence in *F. hepatica* and *E. caproni* EVs (unpublished data).

*Fasciola hepatica* HDM-1/MF6p exhibits biochemical and functional characteristics similar to human defense peptides, particularly CAP18. FhHDM-1 modulates innate cell activation by classical toll-like receptor (TLR) ligands, such as lipopolysaccharide (LPS), indicating its therapeutic potential for autoimmune diseases (65). Furthermore, FhHDM-1 might mitigate the inflammatory response of macrophages to LPS by inhibiting the production of TNFα and IL-1β, as mice treated with a single dose of FhHDM-1/MF6p prior to, or after, bacterial LPS had significantly lower levels of circulating TNFα and IL-1β (64–66). FhHDM-1/MF6p has been recently characterized as a heme-binding protein, suggesting that its role as a heme chaperone that may participate in important physiological processes for the parasite (i.e., heme trafficking and storage). However, it does not seem to act as a primary ligand for LPS (67).

It has been shown that *F. hepatica* ESP prevents type 1 diabetes (T1D) in non-obese diabetic (NOD) mice, which is associated with suppression of IFNγ secretion from auto-reactive T cells, and the switch to IgG1 auto-antibody production (68).

As previously mentioned, recent studies have shown the immunomodulatory effect *H. polygyrus* EVs on a murine model (51), confirming previous results with ESP from the same nematode (41).

Future studies will focus on the potential use of parasitic EVs as therapeutic tools to treat autoimmune disorders and chronic inflammation.

## Concluding Remarks

There has been an increasing number of research publications dealing with the study of EVs and their role in intercellular communication and immunomodulation in the last few years. EVs have been described in parasitic organisms, mostly protozoa, and more recently in helminths. Parasitic protozoa EVs carry virulence factors, immunomodulatory molecules, and nucleic acids. These vesicles have been shown to provide long-lasting protection upon immunization and lethal challenge. Current clinical trials are evaluating the use of helminth secretory products to treat chronic inflammatory and autoimmune diseases. Interestingly, some of the characterized parasitic immunomodulatory proteins have been identified in EVs, raising the intriguing possibility of the therapeutic use of parasitic EVs.

## Conflict of Interest Statement

The authors declare that the research was conducted in the absence of any commercial or financial relationships that could be construed as a potential conflict of interest.

## Footnotes

^1^http://www.who.int/neglected_diseases/diseases/en/

^2^http://www.who.int/mediacentre/factsheets/fs375/en/

^3^http://www.who.int/mediacentre/news/releases/2013/world-malaria-report-20131211/en/

^4^http://www.who.int/mediacentre/factsheets/fs340/en/

^5^http://www.who.int/mediacentre/factsheets/fs259/en/

^6^http://www.cdc.gov/parasites/giardia/epi.html#one
